# Degenerative changes of the mandibular condyle and their relationship with joint space: A CBCT study

**DOI:** 10.4317/jced.63280

**Published:** 2025-10-01

**Authors:** Lourdes Nina-Aguilar, Frederick Ramos-Gómez, Marco Sánchez-Tito

**Affiliations:** 1 School of Dentistry, Faculty of Health Sciences, Universidad Privada de Tacna, Tacna, Peru

## Abstract

**Background:**

This study aimed to identify degenerative changes in the mandibular condyle and to evaluate joint space conditions in sagittal and coronal sections using cone beam computed tomography (CBCT). Additionally, it sought to determine the frequency of these changes relative to sex, age, and side of the temporomandibular joint (TMJ).

**Material and Methods:**

A cross-sectional study was conducted on 88 CBCT scans meeting inclusion criteria. Degenerative changes were assessed, and joint spaces were measured following standardized tomographic protocols. Statistical analysis included chi-square, Student’s t-test, and Mann-Whitney U tests, with significance set at *p* < 0.05.

**Results:**

The sample consisted of 24 males (27.2%) and 64 females (72.7%), with a mean age of 31.2 ± 14.6 years. Erosion (30.6%) and condylar flattening (29.5%) were the most prevalent degenerative changes. No significant differences were found between right and left sides (*p* > 0.05) or between sexes (*p* = 0.445). However, degenerative changes varied significantly with age (*p* = 0.005), with sclerosis, osteophytes, and subchondral cysts more frequent in older adults. Comparison of joint spaces in sagittal and coronal sections revealed no significant differences between condyles with and without degenerative changes (*p* > 0.05).

**Conclusions:**

Degenerative changes in the mandibular condyle were common but did not significantly alter joint space dimensions. Erosion and flattening were the predominant findings, and age was associated with specific changes, while sex and side showed no association. CBCT proved effective for detailed assessment of condylar morphology and joint space.

** Key words:**Temporomandibular joint, Mandibular condyle, Temporomandibular joint disorders, Cone-beam computed tomography.

## Introduction

The temporomandibular joint (TMJ) is a unique structure in the human body, characterized by its anatomy, the complexity of its movements, and the biological processes that support its function [1]. However, when the functional demands placed on the TMJ exceed its ability to adapt, various pathological conditions can occur, leading to what are known as temporomandibular disorders (TMDs) [1-3]. TMDs encompass a group of disorders that can impact the mandible, the masticatory muscles, the TMJ itself, and the surrounding tissues [4]. These disorders are often associated with degenerative changes in the TMJ [5]. Systemic conditions, aging, and hormonal factors may limit the adaptive ability of the TMJ, making it more susceptible to dysfunctional remodeling even with normal physiological biomechanical stresses. [6,7].

The most common degenerative changes in the TMJ primarily affect the mandibular condyle and include flattening, sclerosis, erosion, osteophyte formation, subchondral cysts, ankylosis, and/or loose articular bodies [5]. The prevalence of these changes varies among different populations. Nah [8] reported that in the Korean population, the most frequent degenerative changes were sclerosis (30.2%), superficial erosion (29.3%), and flattening (25.5%). In a study by Massilla Mani and Sivasubramani [9], elderly Indian patients diagnosed with TMD showed a marked prevalence of erosion (56.6%) and joint space narrowing (40%). Rehan *et al*. [10] compared Egyptian patients with rheumatoid arthritis to a control group, finding a high prevalence of flattening (50%) and erosions (35.7%) in the latter group.

Conversely, evidence suggests that degenerative changes in the condyle can lead to a narrowed orirregular TMJ space [11,12]. Findings from magnetic resonance imaging (MRI) indicate that this narrowing, along with changes in TMJ morphology and morphometric parameters, may serve as indicators of disc displacement [13,14]. In this context, the evaluation of bone tissue in the TMJ can be conducted using various imaging techniques, including panoramic radiography, computed tomography (CT), and cone-beam computed tomography (CBCT) [15]. Among these methods, CBCT has emerged as the preferred option, providing diagnostic accuracy comparable to that of CT while avoiding superimposition and structural distortion. Additionally, CBCT offers the benefits of reduced radiation exposure and shorter acquisition time [16].

Therefore, the objective of this study was to identify the presence of degenerative changes in the mandibular condyle and assess the conditions of the joint space in both sagittal and coronal sections using cone beam computed tomography. Additionally, the study aimed to determine the frequency of these degenerative changes in relation to sex, age, and the side of the affected temporomandibular joint.

## Material and Methods

- Study design, statistical power, and ethical considerations

**Figure 1:**
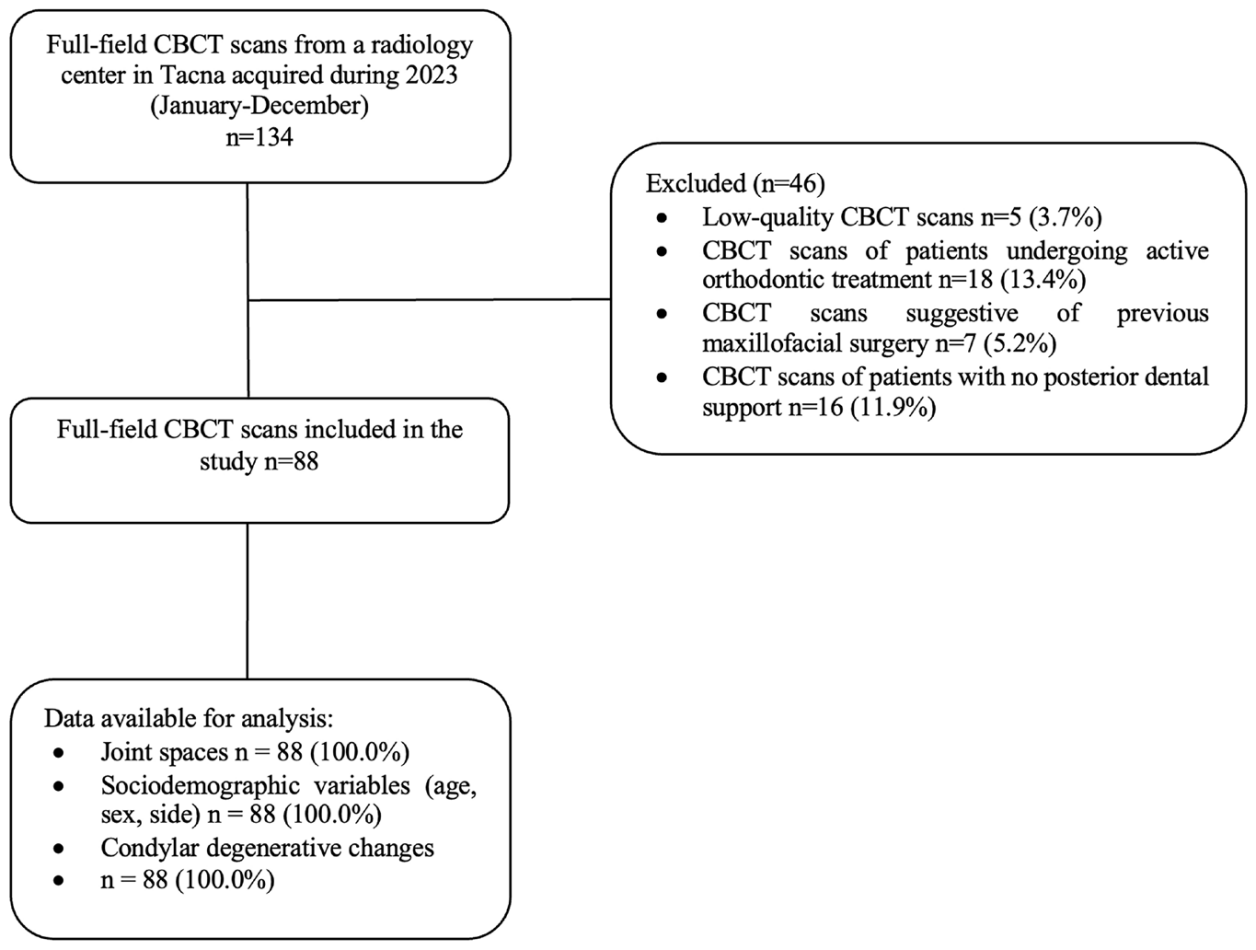
Selection of CBCT scans.

A cross-sectional study was conducted following the STROBE guidelines [17]. All full-field CBCT scans from a radiology center in Tacna, Peru, acquired in 2023, were included if they met the following selection criteria: Cone Beam Computed Tomography (CBCT) scans of patients in occlusion with posterior dental support and with both temporomandibular joints (TMJs) fully visible. We excluded low-quality CBCT scans, those from patients undergoing active orthodontic treatment, and scans showing signs of previous maxillofacial surgery. Ultimately, 88 CBCT scans were evaluated in this study (Fig. 1). The statistical power achieved, based on an expected occurrence proportion of 35% for degenerative changes among the evaluated CBCT scans, was 0.82. This research was approved by the Research Ethics Committee of the Faculty of Health Sciences at the Universidad Privada de Tacna, under registration: FACSA-CEI/080-05-2024. All records were coded to ensure patient anonymity.

- Image Acquisition

For image acquisition, a CBTC system (NewTom GiANO HR) was used, considering a field of view of 16 x 18 cm and a tube voltage of 90 kV. The tube current ranged from 5 to 13 mA, with scan times ranging from 16.8 s to 33.6 s and an exposure time of 10.4 s. The voxel size was 300 μm, ensuring adequate resolution for volumetric assessment of the airways.

- Tomographic analysis

For image analysis, we evaluated the following degenerative bone changes in the mandibular condyle, following characteristics outlined in previous studies based on the Research Diagnostic Criteria for Temporomandibular Disorders (RDC/TMD) [18]:

1. Flattening: This refers to the loss of the rounded contour of the condyle’s articular surface.

2. Erosion: This indicates a loss of continuity along the cortical margin of the condyle.

3. Osteophyte: This involves marginal hypertrophy with sclerotic borders, accompanied by the formation of new bone tissue on the surface of the condyle.

4. Sclerosis: This is characterized by the thickening of the subchondral bone, which appears as a radiopaque area.

5. Subchondral cyst: This is defined as a cavity within the subchondral bone, appearing as a well-demarcated radiolucent area.

**Figure 2:**
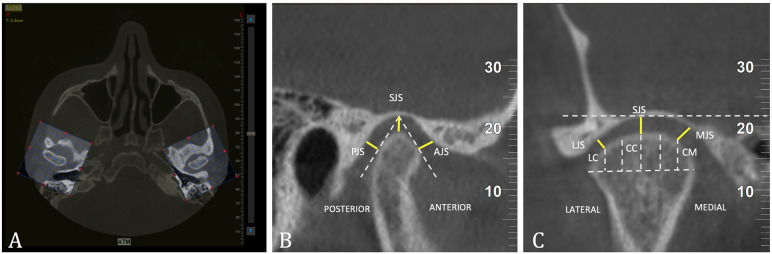
(A) Axial image showing the long axes of the condyles used to create the TMJ images; (B) Measurement of the joint spaces on the sagittal section; (C) Measurement of the joint spaces on the coronal section.

The CBCT scans were exported in Digital Imaging and Communications in Medicine (DICOM) format and digitized using NNT 16.4 software [19]. To obtain sagittal and coronal images of the TMJ, the scan was oriented so that the Frankfurt horizontal plane (Po-Or) was parallel to the floor [20,21]. This orientation was achieved by aligning the axial plane with a line drawn through the highest point of the external auditory meatus and the lowest point of the orbital rim on both the right and left sides [21]. Subsequently, the coronal plane was adjusted using a transportation line [22]. Next, the center and long axes of the condyles were identified from the sagittal and axial views to create the TMJ images [22]. The axial slice thickness was set to 1 mm, and adjustments in direction were made to capture the largest and most distinct images of the condyles for both the right and left joints separately [22], (Fig. 2A).

In the sagittal section, measurements were taken following the method proposed by Gorucu-Coskuner *et al*. [22]. This method involved drawing tangential lines from the highest point of the mandibular fossa to the anterior and posterior surfaces of the condyle (Fig. 2B). Three distinct spaces were defined as follows.

• Anterior Joint Space (AJS): This space is measured from the anterior tangential line to a line that is perpendicular to the mandibular fossa.

• Posterior Joint Space (PJS): This space is measured from the posterior tangential line to a line that is perpendicular to the mandibular fossa.

• Superior Joint Space (SJS): This space is determined by a perpendicular line drawn from the highest point of the mandibular fossa to the most superior surface of the condyle.

For the coronal section, measurements were taken following the recommendations of Ikeda *et al*. [23]. An imaginary horizontal line was drawn at the level of the mandibular fossa. The mediolateral width of the condyle was divided into sextants using lines that were perpendicular to the horizontal line and reached the surface of the condyle, intersecting at the midpoint of the total condylar width. The perpendicular line extending from this midpoint to the condylar surface was designated as the central coronal point (CC). Additionally, the confluence of the first and second medial sextants was labeled the medial coronal point (MC), while the corresponding confluence of the first and second lateral sextants was identified as the lateral coronal point (LC) at the level of the condylar surface. The specific measurements taken corresponded to the following (Fig. 2C).

• Medial Joint Space (MJS): The shortest distance was measured from the medial coronal point (CM) to the mandibular fossa.

• Lateral Joint Space (LJS): The shortest distance was measured from the lateral coronal point (CL) to the mandibular fossa.

• Superior Joint Space (SJS): The shortest distance was measured from the central coronal point (CC) to the mandibular fossa.

To evaluate interobserver reliability, joint space measurements were assessed by both a maxillofacial radiology specialist and the principal investigator. The results obtained by these two evaluators were compared using the Intraclass Correlation Coefficient (ICC). The ICC values ranged from 0.730 to 0.982, indicating a high level of reliability in the measurements taken. Intra-observer reliability was also assessed by repeating measurements at different times, obtaining ICC values between 0.805 and 0.982, which also reflects high reliability.

- Statistical analyses 

Data analysis was conducted using Stata® 19 software (StataCorp LP, College Station, TX, USA). A proportions test was utilized to compare the frequency of degenerative changes between the condyles. The chi-square test determined associations between degenerative changes and the participants’ sex and age. Joint spaces between condyles with and without degenerative changes were compared using the Student’s t-test and the Mann-Whitney U test, depending on the normality of the data, which was assessed using the Shapiro-Wilk test. The significance level for all statistical tests was set at 5%.

## Results

The study sample included 88 records, comprising 24 male patients (27.2%) and 64 female patients (72.7%), with an average age of 31.2 ± 14.6 years.  Table 1 displays the frequency of degenerative changes observed in the mandibular condyle within the sample, with erosion being the most common change (30.6%), followed closely by flattening of the condyle surface (29.5%).

**Figure 3:**
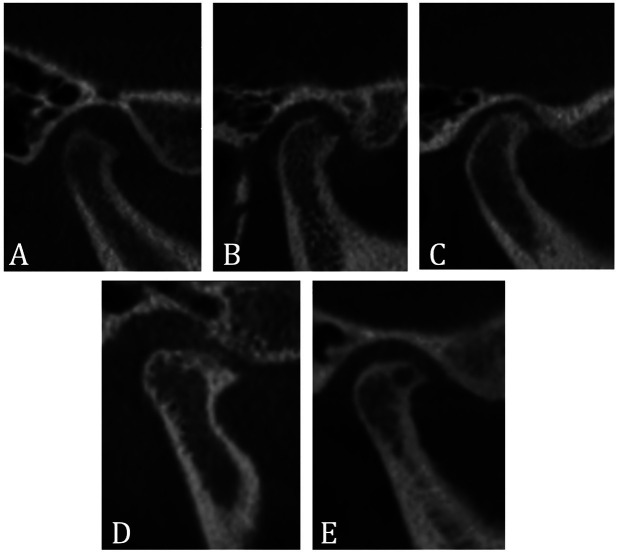
Classification of degenerative changes. (A) Flattening; (B) Erosion; (C) Sclerosis; (D) Osteophyte; (E) Subchondral cyst.

 Table 2 shows the frequency of degenerative changes in the mandibular condyle by affected side. On the right side, condylar flattening was the most frequently observed change (21.6%), whereas, on the left side, erosion predominated (25.0%). No statistically significant differences were found between the right and left sides for any of the degenerative changes (*p* > 0.05). The analysis did not reveal a statistically significant association between the distribution of degenerative changes in the mandibular condyle and gender differences (χ² = 16.1142, *p* = 0.445). However, a statistically significant association was identified between the distribution of these degenerative changes and the patients’ age categories (χ² = 77.2048, *p* = 0.005) ( Table 3). Sclerosis, osteophytes, and subchondral cysts were more prevalent in older adults; while flattening and erosion were more frequent among younger patients (Table 3). Furthermore, the analysis comparing joint spaces in sagittal and coronal sections showed no statistically significant differences (*p* > 0.05) between mandibular condyles exhibiting degenerative changes and those with normal characteristics ( Table 4). Figure 3 shows the main findings of degenerative changes of the mandibular condyle.

## Discussion

Continuous or excessive forces applied to TMJ can result in degenerative changes in the mandibular condyle, which may include a narrowed or irregular joint space [9]. Research indicates that degenerative joint disease can manifest as joint space narrowing, irregularities in the joint space, flattening of the articular surfaces, sclerosis and erosion of the condylar bone surface, the formation of subchondral cysts, and, in later stages, the development of osteophytes [12]. In this study, findings revealed no relationship between degenerative changes in the mandibular condyle and the conditions of the joint space observed in sagittal and coronal sections. These results align with the observations of Görürgöz *et al*. [5] and Tsuruta *et al*., [24] both of whom found no changes in joint space related to the presence or absence of degenerative changes. The discrepancies between these findings and those of other studies may be attributed to differences in the analysis methods used for the TMJ space or variations in the populations studied [25].

Degenerative changes in the temporomandibular joint (TMJ) can indicate different stages of the disease. Erosion is typically linked with the acute or early phases, while flattening, sclerosis, and osteophytes are associated with the late and adaptive phases [5]. In our study, the most common degenerative changes observed were erosion and flattening of the articular cartilage. Koç [11] reported flattening as the most frequent change, with prevalence rates of 36% and 59%, respectively. However, other studies, such as those by Massilla Mani and Sivasubramanian [9], found erosion to be the most prevalent finding at 56.6%. Additionally, Cho and Jung [26] reported erosion as the most prevalent alteration among patients presenting with pain or restricted mouth opening. Such discrepancies across studies may be attribuTable to variations in imaging modalities, population characteristics, or diagnostic criteria [27].

In this study, we were unable to determine any significant differences in the occurrence of degenerative changes between the right and left temporomandibular joints (TMJs). Koç [11] and dos Anjos Pontual *et al*. [28] noted a higher frequency of degenerative changes in the left TMJ, although the difference was not statistically significant. Conversely, Borahan *et al*. [12] reported a higher frequency of degenerative changes on the right side and suggested that this may be linked to individuals’ preferences in masticatory side usage. The disparity in degenerative changes might stem from an uneven distribution of biomechanical stress on the TMJs, influenced by these masticatory preferences [29]. Although bilateral use of the jaw is essential during chewing, it is well established that unilateral predominance in masticatory habits is a common occurrence [30].

This study examined the frequency of degenerative changes in the mandibular condyle based on sex and found that, regardless of the side evaluated, the frequencies were similar between men and women. This suggests that sex is not a significant factor in the development of these changes in the patients studied. However, it is commonly acknowledged that women have a higher prevalence of TMD, and several studies indicate that degenerative changes occur more frequently in female patients [11,28,31]. This difference is primarily attributed to hormonal variations, particularly related to estrogen and prolactin, which may exacerbate the degradation of articular cartilage and bone. Conversely, some researchers have reported no significant differences based on gender [32].

Some research has reported a relationship between degenerative changes and increasing age [12,28]. In our study, we observed that sclerosis, osteophytes, and subchondral cysts were more common in older adults, with significant differences among the various age groups. However, studies by Walewski *et al*. [32] and Crusoe-Rebello *et al*. [33] found no association between age and degenerative changes; instead, they reported significant changes in younger patients. It is important to note that degenerative changes in the TMJ have a multifactorial origin. Factors such as previous trauma, functional overload, parafunctional habits, hormonal alterations, and systemic diseases can contribute to bone changes even in young patients, while older adults may not exhibit such alterations [34].

This study has several limitations that should be considered when interpreting the results. Firstly, the retrospective design did not allow for the control or recording of variables such as the dominant chewing side or history of trauma, which could affect the observed degenerative changes. Secondly, the small sample size limits the generalizability of the results, meaning they should be understood within the study’s narrow scope. Additionally, detailed clinical records were unavailable, preventing direct correlations between the degenerative changes and the patients’ symptoms or clinical conditions. Future research should aim to include additional potential confounding variables to develop a more comprehensive analytical model.

## Conclusions

This study found no significant relationship between degenerative changes in the mandibular condyle and the conditions of the joint space in both sagittal and coronal sections. This indicates that these changes do not consistently impact joint measurements. Erosion was the most common type of degenerative change observed. Other findings, such as sclerosis, osteophytes, and subchondral cysts, were more prevalent in older adults. Additionally, no correlation was identified between sex and the presence of degenerative changes in the mandibular condyle.

## Figures and Tables

**Table 1:** Characteristics of the study sample (n=88).

Characteristics	N (%)
Sex	
Male	24 (27.2)
Female	64 (72.7)
Age (years)	31.2 ± 14.6
Degenerative changes	
Flattening	26 (29.5)
Sclerosis	14 (15.9)
Erosion	27 (30.6)
Osteophyte	13 (14.7)
Subchondral cyst	8 (9.1)
Other	2 (2.3)

† Mean ± Standard deviation. * Percentages may add up to more than 100% because these are multiple-choice data.

**Table 2:** Comparison of the frequency of degenerative changes in the mandibular condyles (n=91).

Degenerative Changes	Condyle		p-value
	Right (n=45)	Left (n=46)	
	n (%)	n (%)	
Flattening	19 (21.6)	11 (12.5)	0.108
Sclerosis	7 (8.0)	9 (10.2)	0.600
Erosion	12 (13.6)	22 (25.0)	0.056
Osteophyte	9 (10.2)	6 (6.8)	0.418
Subchondral cyst	7 (8.0)	4 (4.5)	0.350

† Proportion test.

**Table 3:** Association between degenerative changes of the mandibular condyle and the sex and age group of the participants.

Degenerative Changes	Sex		p-value	Age Group				p-value
	Male (n=24)	Female (n=66)		Young (n=37)	Young adults (n=36)	Mature adults (n=7)	Old adults (n=8)	
	n (%)	n (%)		n (%)	n (%)	n (%)	n (%)	
Flattening	6 (23.1)	20 (76.9)	0.445	9 (34.6)	13 (50.0)	1 (3.8)	3 (11.5)	0.005
Sclerosis	5 (35.7)	9 (64.3)		2 (14.3)	5 (35.7)	2 (14.3)	5 (35.7)	
Erosion	7 (25.9)	20 (74.1)		13 (48.1)	9 (33.3)	2 (7.4)	3 (11.1)	
Osteophyte	4 (30.7)	9 (69.2)		2 (15.2)	4 (30.7)	2 (15.3)	5 (38.4)	
Subchondral cyst	1 (12.5)	7 (87.5)		1 (12.5)	2 (25.0)	1 (12.5)	4 (50.0)	
Other	1 (50.0)	1 (50.0)		1 (50.0)	1 (50.0)	0 (0.0)	0 (0.0)	

†Chi-square test.

**Table 4:** Comparison of the right and left joint spaces in sagittal and coronal sections (mm) according to the presence of degenerative changes.

Tomographic section	Joint space	With changes x ± SD	Without changes x ± SD	p-value
Right sagittal section	AJS	2.1±0.8	2.3 ±0.7	0.240
	PJS	2.2±1.5	1.8±1.4	0.189
	SJS	2.5±1.2	2.6±1.1	0.988
Left sagittal section	AJS	2.1±0.8	2.1±0.6	0.432
	PJS	2.2±1.1	1.9±0.7	0.597
	SJS	2.6±1.1	2.6±0.8	0.687
Right coronal section	LJS	1.6±0.9	1.8±0.6	0.192
	MJS	2.2±1.2	2.3±0.9	0.238
	SJS	2.2±1.0	2.4±0.7	0.057
Left coronal section	LJS	1.9±0.8	1.7±0.6	0.794
	MJS	2.3±1.0	2.4±0.6	0.141
	SJS	2.4±1.1	2.4±0.7	0.380

x̄: mean, SD: standard deviation, AJS: anterior joint space, PJS: posterior joint space, SJS: superior joint space, LJS: lateral joint space, MJS: medial joint space, SJS: superior joint space. †Student t-test, ‡Mann-Whitney U test.

## Data Availability

The datasets used and/or analyzed during the current study are available from the corresponding author.
